# Investigation of Post-Transplant Mental Well-Being in Liver Transplant Recipients with Hepatic Encephalopathy

**DOI:** 10.3390/jcm13113249

**Published:** 2024-05-31

**Authors:** Serdar Saritaş, Sultan Tarlaci, Semra Bulbuloglu, Hüseyin Guneş

**Affiliations:** 1 Department of Medical Biology, Faculty of Medicine, Malatya Turgut Ozal University, 44000 Malatya, Turkey; serdar.saritas@ozal.edu.tr; 2 Division of Neuroscience, Psychology Department, Faculty of Medicine, Uskudar University, 34662 Istanbul, Turkey; sultan.tarlaci@uskudar.edu.tr; 3 Division of Surgical Nursing, Nursing Department, Health Sciences Faculty, Istanbul Aydin University, 34662 Istanbul, Turkey; 4 Division of Surgical Nursing, Nursing Department, Health Sciences Faculty, Bayburt University, 69000 Bayburt, Turkey; huseyingunes@bayburt.edu.tr

**Keywords:** hepatic encephalopathy, liver transplantation, long-term healing

## Abstract

**Objective**: In this study, we aimed to examine the healing trend of hepatic encephalopathy after transplantation surgery in patients with liver failure. **Method**: We conducted this descriptive and cross-sectional study with the participation of liver transplant recipients. A personal information form, the West Haven Criteria (WHC), the Warwick-Edinburgh Mental Well-Being Scale (WEMWBS), and the Richmond Agitation Sedation Scale (RASS) were used for data collection. The data were analyzed using Chi-squared tests, ANOVA, and paired-samples *t*-tests. **Results**: As time progressed after liver transplantation, hepatic encephalopathy stages regressed (*p* < 0.01). We found that liver transplant recipients with end-stage hepatic encephalopathy were mostly within the first 6 months after transplantation, while patients with first-stage hepatic encephalopathy had received liver transplants more than 2 years ago (*p* < 0.01). **Conclusions**: The results of our study revealed that hepatic encephalopathy stages regressed after transplantation, but there was no complete recovery. This highlights the need to develop new treatment strategies other than liver transplantation for the treatment of hepatic encephalopathy.

## 1. Introduction

Hepatic encephalopathy, which is a significant complication developing in relation to severe acute or chronic liver failure, leads to disorders in cognitive and consciousness levels and motor activity [1]. In acute and chronic liver failure cases, portosystemic shunting and cirrhosis play an active role in the etiology of hepatic encephalopathy [2]. Hepatic encephalopathy is a prognostic criterion in chronic liver problems and serves as an indicator for the consideration of liver transplantation surgery [3,4]. People with chronic liver disease were reported to have schizophrenia- or mania-like behaviors, paraplegia, ganglion disorders, consciousness disorders or muscle spasms, localized or generalized cortical diseases, and symptoms of parkinsonism [5,6]. Among various symptoms, cognitive symptoms are the earliest to develop in hepatic encephalopathy [1]. Clinicians should look out for deterioration in visuoconstruction and psychomotor abilities and screen for hepatic encephalopathy using psychometric tests [1,7].

The Model for End-Stage Liver Disease (MELD) scoring system is used to determine priority for liver transplant surgery in chronic liver failure cases [8]. The MELD scoring system uses serum bilirubin, serum creatinine, the international normalized ratio (INR) for prothrombin time, and albumin values to predict post-transplant survival [9]. Liver patients with a MELD score above 30 are more likely to be admitted to the intensive care unit (ICU) and stay in the ICU after liver transplantation, have a higher dose of vasopressor therapy before transplantation, and require more transfusion in the intraoperative period. Additionally, these patients need more mechanical ventilator support and renal replacement therapy after transplantation [10]. The higher the pre-transplant MELD score, the higher the probability of graft rejection and failure in the post-operative period [11,12]. The Child–Pugh scoring system also considers the presence of hepatic encephalopathy in addition to examining parameters similar to those in MELD scoring [13].

Hepatic encephalopathy is graded using the West Haven Criteria (WHC) [14]. Triggering factors can be listed as gastrointestinal bleeding, types of infection, high-dose diuretic use, constipation, use of psychiatric drugs, fluid-electrolyte disorders, dehydration, and dietary disorders [1]. In the treatment of hepatic encephalopathy, there are practices such as infection treatment, interventions to reduce ammonia concentrations (use of lactulose and similar disaccharides), testing for and treating Helicobacter pylori infection, flumazenil, benzodiazepine blockade, and preferably haloperidol usage. Hepatic encephalopathy is an indicator of poor prognosis in liver disease. After the first attack, its 1-year survival rate is 42%, and its 3-year survival rate is 23% [15]. Therefore, liver transplantation should be considered in patients with hepatic encephalopathy, and the patient should be evaluated by assessing their need for transplantation.

The number of studies in the literature regarding the recovery of cognitive disorders and psychomotor abilities in the post-operative period of patients who have had hepatic encephalopathy before transplantation surgery is limited. We believe that restoring the neuropsychiatric functions of patients will improve their compliance with post-transplantation care and treatment, as well as their survival. In this study, we aimed to examine the trend in the treatment of hepatic encephalopathy after transplantation surgery in patients with liver failure.

## 2. Materials and Methods

This study was carried out as a descriptive, cross-sectional, and single-center study to investigate the trend of long-term healing in hepatic encephalopathy after liver transplantation. Our study was conducted with the participation of liver transplant patients at an organ transplantation institute of a research and training hospital.

### 2.1. Design and Participants

After obtaining ethics committee approval, we conducted this study with the participation of 306 liver transplant patients who were diagnosed with hepatic encephalopathy in the pre-transplant stage based on the West Haven Criteria. Patients were included using the purposive sampling method. Patient information was obtained from the electronic patient records of the hospital where the study was conducted. Liver transplant patients were contacted by phone and invited to the hospital. In a private interview room, the researchers interviewed each patient individually for 20 min. The sociodemographic and surgery-related information of the patients was collected using a personal information form. The Richmond Agitation Sedation Scale (RASS) scores of the patients on the 7th day after liver transplantation were recorded by looking at their electronic patient records. Similarly, according to the West Haven Criteria, the grade before liver transplantation was recorded using electronic physician notes. The researchers and the assigned physician cooperated in determining the post-transplant grade. The Warwick-Edinburgh Mental Well-Being Scale (WEMWBS) is a self-assessment tool. The patients were asked to make two separate evaluations, considering their pre- and post-transplant status for WEMWBS. The inclusion and exclusion criteria of the study are listed below.

### 2.2. Inclusion Criteria

- Receiving a liver transplant in the hospital where the study was conducted;
- Being diagnosed with hepatic encephalopathy before liver transplantation;
- Being 18 years old or older;
- Not having any communication barriers, and conscious patients;
- Agreeing to participate in the study.

### 2.3. Exclusion Criteria

- Not receiving a liver transplant or receiving a liver transplant at a hospital other than the hospital where the study was conducted;
- Not having a diagnosis of hepatic encephalopathy before liver transplantation;
- Being under 18 years of age, not speaking Turkish, or having a communication barrier, unconscious and intubated patients;
- Refusing to participate in the study.

### 2.4. Data Collection Tools

A personal information form, the West Haven Criteria (WHC), the Warwick-Edinburgh Mental Well-Being Scale (WEMWBS), and the Richmond Agitation Sedation Scale (RASS) were used for data collection. WEMWBS and RASS were applied as pretest and posttest to all recipients before and after liver transplantation.

### 2.5. Personal Information Form

The researchers developed the form after expert consultation. The form included questions on the sociodemographic characteristics of the patients (e.g., age, sex, marital status, education level) and their surgery-related information.

### 2.6. West Haven Criteria (WHC)

The diagnosis of hepatic encephalopathy, which is a serious complication causing significant morbidity and mortality in patients with acute or end-stage liver failure [15], is made by neurological findings obtained in clinical examinations. This diagnosis is based on the state of consciousness of the patient, their intellectual functions, and behavioral changes, as well as signs of neuromuscular abnormalities. The West Haven Criteria used in the diagnosis [16] and staging of hepatic encephalopathy involves a classification of patients into four grades [17]. Based on the severity of clinical findings, the hepatic encephalopathy grades can be divided into two main groups as overt hepatic encephalopathy (WHC Grades 2–4) and covert hepatic encephalopathy (minimal hepatic encephalopathy or WHC Grade 1) [17,18]. Covert hepatic encephalopathy is considered the preclinical stage of overt hepatic encephalopathy [18]. While overt hepatic encephalopathy refers to the presence of clear neurological findings, covert hepatic encephalopathy refers to a clinical diagnosis that can only be made by specialized tests [16].

### 2.7. Warwick-Edinburgh Mental Well-Being Scale (WEMWBS)

WEMWBS was developed by Tennant et al. (2007) [19] in the United Kingdom to measure psychological well-being. Keldal (2015) [20] adapted the scale to Turkish. The scale consists of 14 items. A minimum of 14 and a maximum of 70 points can be obtained from the 5-point Likert-type scale. Higher scores are interpreted as higher levels of mental well-being. The response options for each item are 1 = None of the time, 2 = Rarely, 3 = Some of the time, 4 = Often, 5 = All of the time. All items on the scale are positive statements, so there is no inversely scored item. The Cronbach’s alpha coefficient of the scale was reported as 0.89. In this study, this coefficient was found to be 0.90.

### 2.8. Richmond Agitation Sedation Scale (RASS)

Some researchers use RASS to identify hepatic encephalopathy, a subtype of delirium. Patients with hepatic encephalopathy may be hyperactive or hypoactive. The RASS score is between +1 and +4 in hyperactive individuals and between −3 and 0 in hypoactive individuals [21]. This scale is frequently used in research settings and clinical practice when monitoring patients for diagnosis [22,23]. The Turkish version has been used in clinical and research areas for a long time in Turkey, but a study to test its validity and reliability was performed only recently by Sılay and Akyol [24].

### 2.9. Classification of Ammonia Levels

Serum ammonia levels are a controversial issue in defining hepatic encephalopathy. There is no consensus on the rating of ammonia levels. We rated ammonia levels in line with common practice and expert opinion. Accordingly, the range for normal people is from 5 to 53 μmol/L, for slightly elevated people it is from 54 to 72 μmol/L, for high ammonia level it is from 73 to 80 μmol/L, for very high ammonia level it is from 81 to 90 μmol/L. For extremely high, the range from 91 to 100 μmol/L was accepted [25,26,27,28,29].

### 2.10. Data Analysis

After the data were coded by the researchers, data analysis was performed by using Statistical Package for the Social Sciences (SPSS) 25.0 IBM (Armonk, NY, USA). Descriptive statistics were used in the analyses. Prior to the analyses, the normality of the distribution of the data was tested using the Kolmogorov–Smirnov test. Chi-squared test, one-way analysis of variance (ANOVA), and paired-samples *t*-test (used to find the significance of the difference between the arithmetic means of two related groups) were performed to determine the relationships between scale scores and descriptive characteristics. The relationships between the categorical variables were examined using the Phi and Cramer’s V correlation tests. Cronbach’s alpha coefficient was used to determine the reliability levels of the scales. Post hoc comparisons were used to identify the sources of significant differences. The results were evaluated in a 95% confidence interval and at a significance level of *p* < 0.05.

### 2.11. Ethical Considerations

Prior to the study, ethical approvals were obtained from Turgut Ozal Medical Center and Inonu University Ethics Committee (Date: 11 January 2022, Decision No: 2022/2929). The patients were informed about the study in accordance with the principles of the Declaration of Helsinki, and their approval for the voluntary information form was obtained. The patients who agreed to participate were included in the study after their written consent was obtained.

## 3. Results

Table 1 shows the distribution of the patients based on their personal characteristics and mean WEMWBS scores. We found that 37.3% of the 306 patients were between 51 and 65 years old, 57.5% of them were male, and 35% were high school graduates. It was determined that 69% of the patients had normal serum ammonia levels before liver transplantation. While 35.9% of the patients had undergone liver transplant surgery within the last 6 months, 33.7% had undergone their surgeries between the last 1 year and 6 months.

Table 2 shows the distribution of the patients based on their health-related characteristics and mean WEMWBS and RASS scores. We determined that 53.3% of the patients had diabetes mellitus after liver transplantation, and biliary-pancreatic disease was found in 80.1% before transplantation. According to WHC, we found that 24.5% and 35.3% of the pre- and post-transplant liver patients were WHC Grade 1, and 32.4% and 28.1% were WHC Grade 2, respectively. According to their RASS scores on the seventh day after liver transplantation, 25.8% of the patients were ill-tempered, and 21.9% were sleepy based on the information obtained from their patient files. The mean WEMWBS score of the patients was 26.40 ± 6.35 (min: 16, max: 54). The WEMWBS scores of those who had normal ammonia levels before liver transplantation and those who had undergone transplantation surgery more than 2 years ago were found to be significantly higher (Table 2).

Table 3 presents the mean hepatic encephalopathy grades of the patients before and after their liver transplantations, as well as the results of the comparisons of these grades. The mean pre- and post-transplant hepatic encephalopathy grades of the patients were found, respectively, as 2.38 ± 1.06 and 2.17 ± 1.08, and the difference between these two values was statistically significant (t = 8.891, *p* = 0.000).

Table 4 shows the results of the correlation analysis of hepatic encephalopathy stages before and after liver transplantation. Accordingly, hepatic encephalopathy grades regressed as time progressed after liver transplantation (*p* = 0.000, Phi = 1.587 and Cramer’s V = 0.916). However, the patient with the best recovery regressed to grade 1, and none of them had a full recovery.

**Figure 1:**
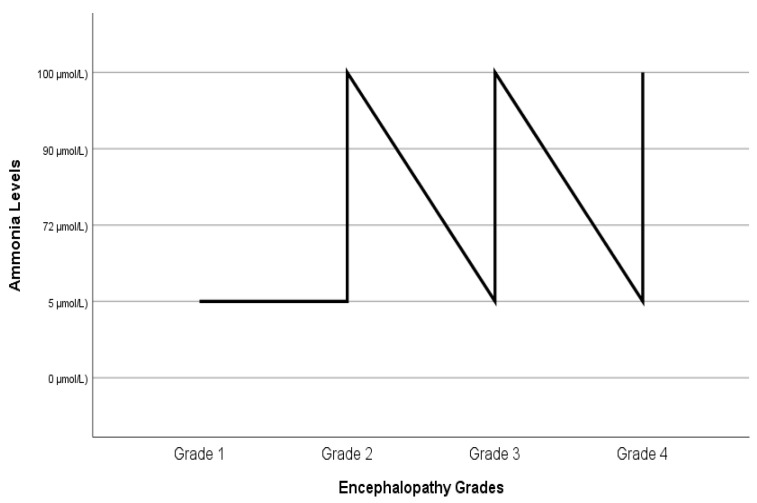
Comparison of ammonia levels and grades of hepatic encephalopathy before liver transplantation.

Figure 1 shows the comparison of serum ammonia levels and hepatic encephalopathy grades before liver transplantation. Some patients with grade 1 and 2 encephalopathy appear to have high ammonia levels, while some patients with grade 3 and 4 encephalopathy appear to have normal ammonia levels.

**Figure 2:**
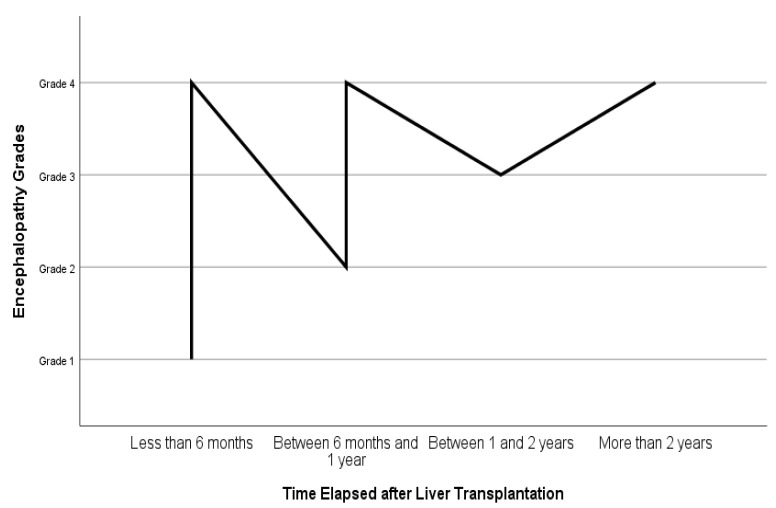
Time elapsed after liver transplantation and grades of hepatic encephalopathy.

In Figure 2, the relationship between the time elapsed after liver transplantation and the grade of encephalopathy is illustrated. Accordingly, the patients with grade 4 hepatic encephalopathy were mostly within the first 6 months after their transplantation, while those with grade 1 hepatic encephalopathy had received liver transplants more than 2 years ago (*n* = 306).

## 4. Discussion

Hyperammonemia is widely accepted as the most important indicator of hepatic encephalopathy prior to liver transplantation [30]. This is one of the two most important obstacles to the treatment and care of patients with normal serum ammonia levels for hepatic encephalopathy. The second obstacle is the awareness that hepatic encephalopathy is a subtype of delirium [31,32]. This situation causes the exclusion of drug and non-drug interventions used in the diagnosis, prevention, treatment, care, and management of delirium in the management of hepatic encephalopathy. Today, these practices are not used in the management of encephalopathy, and this situation leads to the need for further research.

Conflicting results in the literature limit ammonia as an indicator associated with the presence of hepatic encephalopathy [2,29,33]. In addition, it is recommended that the change in ammonia level coincides with the MELD scoring in the treatment and management of hepatic encephalopathy [29]. Even if the ammonia level normalizes, it cannot be assumed that the psychometric situation has improved. Similarly, absence of hepatic encephalopathy is not guaranteed in liver failure patients with good ammonia levels. Patients with normal pre-transplant ammonia levels were also included in the sample of our study due to poor psychometric test results. In this regard, the findings of this study are an important source of information. In this study, as the ammonia level increased before liver transplantation, mental well-being decreased.

Confusion and hepatic encephalopathy may occur in patients with end-stage liver failure. One of the reasons for this is the accumulation of manganese in the brain. Computed tomography (CT) does not have a place in detecting manganese accumulation, and the diagnosis is made with the help of magnetic resonance imaging (MRI). This accumulation is observed as an increase in signal intensity in T1 mapping sequences in the lentiform nuclei, mesencephalon, and anterior part of the pituitary gland. These findings disappear after liver transplantation as the new liver tissue becomes functional. Additionally, similar MRI findings can be seen in Wilson’s disease, manganese poisoning, and patients receiving total parenteral nutrition for a long time [34]. In our study, the rate of patients whose liver problems continued after liver transplantation was 8.2%, and their mental well-being was the poorest among all patients (*p* = 0.014). The findings of our study supported the results in the relevant literature.

In the literature, some studies have shown that the severity of hepatic encephalopathy in the pre-transplant period affects awareness and alertness after transplantation, but the findings of these studies have not revealed definitive results [35,36,37]. To the best of our knowledge, no study has yet addressed the trend of long-term healing in hepatic encephalopathy cases after liver transplantation. It has been reported that it takes more than 5 days for a patient to regain consciousness after a transplant in fourth-stage hepatic encephalopathy cases [37,38]. In our study, based on their RASS scores, only 9% of the patients were awake and calm, 25.8% of them were ill-tempered, and 21.9% were sleepy after liver transplantation. Furthermore, it was discovered in our study that the patients with normal and slightly elevated ammonia levels before transplantation had better post-transplant mental well-being levels.

We concluded that the encephalopathy stages of the patients regressed as time passed after liver transplantation (*p* = 0.000, Phi = 1.587, and Cramer’s V = 0.916). Additionally, all patients who had undergone transplant surgery more than 2 years ago were in the first-stage hepatic encephalopathy category. Although this situation was pleasing in comparison to transplant patients with advanced hepatic encephalopathy, it was not a satisfying result in that the patients who had undergone transplant surgery more than 2 years ago had not fully recovered. Similar research has found that the presence of a persistent shunt, the development of ischemic brain injury during the intraoperative process, and/or permanent brain damage prior to transplantation all cause irreversible hepatic encephalopathy in the post-transplant period [39,40,41,42]. The findings of our study supported the results in the relevant literature.

The conditions that were acknowledged as limitations of our study were not performing MRI for all patients due to cost constraints, not examining parameters such as serum ammonia and other vitamin deficiency conditions that could cause hepatic encephalopathy, and only using psychometric tests to interpret the results. Our study is a single-center study, so our results may not be generalized to the population. None of the data collection tools we used in our study had been developed specifically for hepatic encephalopathy. Therefore, the exact etiology of encephalopathy could not have been determined. Factors such as manganese accumulation in the brain, prolonged stay in the intensive care unit, cerebrovascular disease, or psychiatric disease may have played a role in the etiology of post-transplant encephalopathy development. All these factors were accepted as limitations.

## 5. Conclusions

The complex nature of hepatic encephalopathy complicates patient follow-up in the peri-operative and long-term post-operative periods. The inability to completely control hepatic encephalopathy in patients who have undergone liver transplantation is due to both knowledge gaps in the literature and a lack of awareness among healthcare practitioners. It is of great importance to understand hepatic encephalopathy and deal with the parameters affecting it in many respects such as physiological, social, psychological, and surgery-related contexts. Our study is the only study to investigate the long-term trend of hepatic encephalopathy in liver transplant patients. Our data revealed that hepatic encephalopathy regressed after transplantation, albeit not completely. This highlights the need to develop new treatment strategies after liver transplantation for the complete resolution of hepatic encephalopathy.

## Figures and Tables

**Table 1:** Distribution of liver recipients according to their personal characteristics and mean WEMWBS scores (*n* = 306).

Characteristics			WEMWBS
Age	*n*	%	Mean ± SD
20–35 years	11	3.6	26.9 ± 6.46
36–50 years	98	32	28.31 ± 6.72
51–65 years	114	37.3	26.57 ± 6.31
66 years or older	83	27.1	25.39 ± 6.63
**Test and *p*-value**			F = 1.052
			*p* = 0.397
**Sex**	***n***	**%**	**Mean ± SD**
Female	130	42.5	25.93 ± 6.18
Male	176	57.5	26.75 ± 6.46
**Test and *p*-value**			**χ^2^** = 1.304
			*p* = 0.145
**Education Level**	***n***	**%**	**Mean ± SD**
Literate with no Formal Degree	107	35	27.51 ± 7.48
Primary School	37	12	25.64 ± 5.76
High School	107	35	25.52 ± 5.85
University and above	55	18	26.47 ± 4.9
**Test and *p*-value**			F = 1.641
			*p* = 0.211
**Ammonia levels before liver transplantation**	***n***	**%**	**Mean ± SD**
Normal (from 5 to 53 μmol/L) (1)	211	69	32.28 ± 6.12
Slightly elevated (from 54 to 72 μmol/L) (2)	41	13.4	27.12 ± 5.71
High (from 73 to 80 μmol/L) (3)	25	8.2	26.26 ± 6.1
Very high (from 81 to 90 μmol/L) (4)	22	7.2	26.63 ± 6.03
Extremely high (from 91 to 100 μmol/L) (5)	7	2.3	25.58 ± 6.65
**Test and *p*-value**			F = 1.118
			***p*** = 0.031 *
			**Post hoc** 1 > 2 > 3,4,5
**Time after liver transplant**	***n***	**%**	
6 months (1)	110	35.9	25.27 ± 5.75
Between 6 months and 1 year (2)	103	33.7	26.28 ± 6.38
Between 1 and 2 years (3)	49	16	27.51 ± 5.21
More than 2 years (4)	44	14.4	28.29 ± 6.41
**Test and *p*-value**			F = 1.252
			***p*** = 0.016 *
			**Post hoc** 1 < 2 < 3 < 4

χ^2^, Chi-square test; F, One-way analysis of variance (ANOVA); * *p* < 0.05, SD, Standard Deviation.

**Table 2:** Distribution of liver recipients according to their health-related characteristics and mean WEMWBS scores (*n* = 306).

Characteristics			WEMWBS
Problems after Liver Transplantation *	*n*	%	Mean ± SD
DM (1)	163	53.3	28.73 ± 6.36
Kidney disorders (2)	15	4.9	26.89 ± 6.43
Hepatocellular Carcinoma (3)	48	15.6	26.92 ± 6.53
Thyroid disorders (4)	21	6.9	27.29 ± 6.68
Liver disorders (5)	25	8.2	24.88 ± 6.65
Peripheral neurotoxicity (6)	69	22.5	27.37 ± 6.2
**Test and *p*-value**			F = 1.740
			***p*** = 0.014 *
			Post hoc 5 < 1,2,3,4
**Concomitant diseases before liver transplantation**	***n***	**%**	**Mean ± SD**
None	48	15.7	37.07 ± 6.22
Bile-pancreatic disorders	245	80.1	28.46 ± 5.78
Diabetes mellitus (DM)	53	17.3	25.54 ± 6.73
Chronic obstructive pulmonary disease (COPD)	39	12.7	27.14 ± 3.27
Chronic Renal Failure (CRF)	41	13.4	24.57 ± 6.6
Cardiovascular Disease (CVD)	37	12.1	23.84 ± 6.11
Asthma	27	8.8	24.66 ± 5.67
**Test and *p*-value**			F = 1.048
			*p* = 0.403
**Grades of Hepatic Encephalopathy Based on the West Haven Criteria**			
**Pre-transplant phase**	***n***	**%**	**Mean ± SD**
Grade 1 (1)	75	24.5	31.12 ± 5.36
Grade 2 (2)	99	32.4	27.52 ± 4.48
Grade 3 (3)	71	23.2	24.41 ± 4.37
Grade 4 (4)	61	19.9	-
**Test and *p*-value**			F = 0.906
			***p*** = 0.011 *
			Post hoc 1 > 2 > 3
**Grades of Hepatic Encephalopathy Based on the West Haven Criteria**			
**Post-transplant phase**	***n***	**%**	**Mean ± SD**
Grade 1 (1)	108	35.3	33.40 ± 6.35
Grade 2 (2)	86	28.1	28.72 ± 5.32
Grade 3 (3)	62	20.3	26.36 ± 6.3
Grade 4 (4)	50	16.3	-
**Test and *p*-value**			F = 1.083
			***p*** = 0.026 *
			Post hoc 1 > 2 > 3
**RASS Scores on Day 7 after Liver Transplantation**	***n***	**%**	**Mean ± SD**
Awake and calm (1)	29	9.5	29.31 ± 5.67
Sleepy (2)	67	21.9	27.67 ± 6.24
Lightly sedated (3)	25	8.2	25.44 ± 6.11
Moderately sedated (4)	28	9.2	25.25 ± 6.67
Deeply sedated (5)	31	10.1	24.41 ± 6.49
Uneasy (6)	21	6.9	23.23 ± 6.08
Agitated (7)	12	3.9	24.33 ± 6.85
Highly agitated and aggressive (8)	11	3.6	25.90 ± 6.49
Ill-tempered (9)	79	25.8	25.59 ± 6.2
Unconscious (10)	3	0.9	25.40 ± 5.35
**Test and *p*-value**			F = 2.964
			***p*** = 0.000 **
			Post hoc 1,2 > 3,4,5,6,7,8,9,10
**WEMWBS Score (Mean ± SD)**	**Number of Items**	**Score Range**	**Min.–Max**
26.40 ± 6.35	14	14–70	16–54

Χ^2^, Chi-Squared test; F, One-way analysis of variance (ANOVA); * *p* < 0.05, ** *p* < 0.01; SD, Standard Deviation.

**Table 3:** Intragroup comparison of pre- and post-transplant mean hepatic encephalopathy grades (*n* = 306).

Grades of Hepatic Encephalopathy		Intergroup Comparison	
Phase	Mean ± SD	t	Sig. (2-Tailed)
Pre-transplant	2.38 ± 1.06	8.981	0.000 **
Post-transplant	2.17 ± 1.08		

t: Paired-samples *t*-test, ** *p* < 0.01.

**Table 4:** Crosstabulation of encephalopathy grades according to West Haven Criteria with the time spent after liver transplantation (*n* = 306).

					Encephalopathy Grades				Total
					Grade 1	Grade 2	Grade 3	Grade 4	
**Time after liver transplant**	More than 2 years			**Count**	108	0	0	2	110
				Expected Count	38.8	30.9	22.3	18.0	110.0
				% within Transplant time	98.2%	0.0%	0.0%	1.8%	100.0%
	Between 1 and 2 years			Count	0	86	13	4	103
				Expected Count	36.4	28.9	20.9	16.8	103.0
				% within Transplant time	0.0%	83.5%	12.6%	3.9%	100.0%
	Between 6 months and 1 year			Count	0	0	49	0	49
				Expected Count	17.3	13.8	9.9	8.0	49.0
				% within Transplant time	0.0%	0.0%	100.0%	0.0%	100.0%
	Less than 6 months			Count	0	0	0	44	44
				Expected Count	15.5	12.4	8.9	7.2	44.0
				% within Transplant time	0.0%	0.0%	0.0%	100.0%	100.0%
Total				Count	108	86	62	50	306
				Expected Count	108,0	86.0	62.0	50.0	306.0
				% within Transplant time	35.3%	28.1%	20.3%	16.3%	100.0%
**Symmetric Measures**									
			Value	Approximate Significance					
Nominal by Nominal		Phi	1.587	0.000 **					
		Cramer’s V	0.916	0.000 **					
N of Valid Cases			306						

*p* < 0.01 **; Expected Count→Percentage of sample required to reach statistical insignificance.

## Data Availability

The original contributions presented in the study are included in the article, further inquiries can be directed to the corresponding author.
